# The Acute, Short-, and Long-Term Effects of Endurance Exercise on Skeletal Muscle Transcriptome Profiles

**DOI:** 10.3390/ijms25052881

**Published:** 2024-03-01

**Authors:** Thomas Beiter, Martina Zügel, Jens Hudemann, Marius Schild, Annunziata Fragasso, Christof Burgstahler, Karsten Krüger, Frank C. Mooren, Jürgen M. Steinacker, Andreas M. Nieß

**Affiliations:** 1 Department of Sports Medicine, Medical Clinic, Eberhard-Karls-University of Tübingen, 72076 Tübingen, Germany; thomas.beiter@med.uni-tuebingen.de (T.B.); annunziata.fragasso@med.uni-tuebingen.de (A.F.); christof.burgstahler@med.uni-tuebingen.de (C.B.); 2 Department of Sport and Rehabilitation Medicine, University of Ulm, 89075 Ulm, Germanyjuergen.steinacker@uni-ulm.de (J.M.S.); 3 Department of Exercise Physiology and Sports Therapy, University of Gießen, 35394 Gießen, Germanykarsten.krueger@sport.uni-giessen.de (K.K.); 4 Department of Medicine, Faculty of Health, University of Witten/Herdecke, 58455 Witten, Germany; frank.mooren@uni-wh.de

**Keywords:** skeletal muscle transcriptomics, gene expression, exercise physiology, skeletal muscle, endurance training, acute exercise, training status

## Abstract

A better understanding of the cellular and molecular mechanisms that are involved in skeletal muscle adaptation to exercise is fundamentally important to take full advantage of the enormous benefits that exercise training offers in disease prevention and therapy. The aim of this study was to elucidate the transcriptional signatures that distinguish the endurance-trained and untrained muscles in young adult males (24 ± 3.5 years). We characterized baseline differences as well as acute exercise-induced transcriptome responses in *vastus lateralis* biopsy specimens of endurance-trained athletes (ET; n = 8; VO_2_max, 67.2 ± 8.9 mL/min/kg) and sedentary healthy volunteers (SED; n = 8; VO_2_max, 40.3 ± 7.6 mL/min/kg) using microarray technology. A second cohort of SED volunteers (SED-T; n = 10) followed an 8-week endurance training program to assess expression changes of selected marker genes in the course of skeletal muscle adaptation. We deciphered differential baseline signatures that reflected major differences in the oxidative and metabolic capacity of the endurance-trained and untrained muscles. SED-T individuals in the training group displayed an up-regulation of nodal regulators of oxidative adaptation after 3 weeks of training and a significant shift toward the ET signature after 8 weeks. Transcriptome changes provoked by 1 h of intense cycling exercise only poorly overlapped with the genes that constituted the differential baseline signature of ETs and SEDs. Overall, acute exercise-induced transcriptional responses were connected to pathways of contractile, oxidative, and inflammatory stress and revealed a complex and highly regulated framework of interwoven signaling cascades to cope with exercise-provoked homeostatic challenges. While temporal transcriptional programs that were activated in SEDs and ETs were quite similar, the quantitative divergence in the acute response transcriptomes implicated divergent kinetics of gene induction and repression following an acute bout of exercise. Together, our results provide an extensive examination of the transcriptional framework that underlies skeletal muscle plasticity.

## 1. Introduction

Skeletal muscle is the most abundant tissue in the human body. It gives us posture, protects our bones, and allows our bodies to move. But skeletal muscle is also centrally important in maintaining metabolic and physiological homeostasis. It serves as a regulator of interorgan crosstalk for energy and protein metabolism, exhibits immunobiological properties, and has a profound impact on the central and peripheral nervous system [1,2,3]. To adequately cope with environmental and physiological challenges, skeletal muscle possesses the unique ability to change its size, structural composition, as well as metabolic properties. In this way, specific exercise regimes can be exploited to provoke pronounced adjustments in muscular force, endurance capacity, and/or contractile velocity [4,5]. Functional adaptations are brought about by qualitative and quantitative changes in fiber size and composition that become manifest in a specific assemblage of myofibrillar protein isoforms, a rearrangement of metabolic pathways, and altered Ca^2+^ handling characteristics [6,7]. Such adaptive remodeling of skeletal muscle arises from the cumulative effect of frequently repeated exercise bouts that acutely challenge systemic and local homeostasis at the metabolic, structural, and inflammatory levels. Surprisingly, the mechanistic framework that links these acute exercise-provoked homeostatic perturbations to long-term training adaptations is largely unknown. Despite much effort in the field, there still is no comprehensive understanding of how single-exercise stimuli should become memorized and how subsequent adaptive reprogramming of myofibrillar gene expression and signaling pathways is accomplished [8,9,10].

Strong epidemiological and emerging clinical evidence shows that habitual physical activity is associated with multiple health outcomes [11]. Understanding the molecular mechanisms that govern skeletal muscle plasticity has the potential to broaden fundamental biological knowledge. This may provide a basis for discovering novel strategies and targets for disease prevention and treatment of dysregulated skeletal muscle physiology in various clinical conditions.

High-throughput methods in the fields of transcriptomics, proteomics, and metabolomics provide valuable data compilations to gain insight into the complex signaling pathways that coordinately act to initiate and shape metabolic and structural remodeling in human skeletal muscle in response to acute exercise and long-term training stimulation [12,13,14,15,16,17,18,19,20,21,22,23]. Using a label-free quantitative proteomic approach, our groups could recently decipher various metabolically active proteins with training status-dependent abundances and differential acute response patterns in human *vastus lateralis* muscle [21]. Likewise, through the use of a multi-analyte profiling strategy, we could provide a list of differentially affected circulating mediators with diverse metabolic and immunological properties in plasma samples of endurance-trained and sedentary individuals in response to intensive cycling exercise [24]. To extend these results to the transcriptional level, we further exploited these study protocols to determine the general impact of strength and endurance exercise on skeletal muscle transcriptome profiles [25].

Here, we now provide an in-depth analysis of training status-dependent gene expression signatures in *vastus lateralis* muscle from young endurance-trained (ET) and untrained sedentary individuals (SED) under resting conditions as well as in response to acute exercise. To interpret the biological relevance of observed differential gene expression patterns, microarray data were analyzed for functional and regulatory gene signatures. Expression levels of selected target genes were further exploited to assess the adaptational changes of the untrained muscle in the course of a standardized 8-week endurance training intervention.

## 2. Results

### 2.1. Study Design

**Figure 1:**
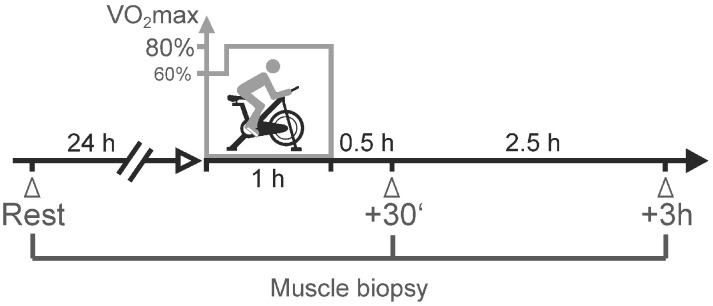
Schematic illustration of the acute exercise setup. Biopsies were obtained from the *vastus lateralis* muscle of the right leg. Baseline expression (rest) was determined from samples taken 24 h before the acute exercise experiment. Exercise consisted of 10 min of moderate warm-up cycling and 50 min of strenuous cycling exercise adjusted in its intensity to 80% of the individual oxygen uptake capacity (VO_2_max). Acute exercise-provoked expression profiles were evaluated from biopsy samples taken at 30 min (+30′) and at 3 h (+3 h) after the cycling exercise.

Healthy, young male volunteers were recruited in this study and categorized based on their physical activity history and VO_2_max levels into untrained sedentary individuals (SED; n = 8) and endurance-trained individuals (ET; n = 8) (see the Section 4 for details). All study participants followed an acute exercise protocol that consisted of 60 min of high-intensity cycling exercise on a cycle ergometer at a main power requiring 80% of the individual VO_2_max (including 10 min of initial warm-up cycling). Baseline microbiopsy samples of the *vastus lateralis* muscle were obtained 24 h before the acute exercise protocol was started. Post-exercise biopsies were taken at 30 min and at 3 h after cessation of acute cycling exercise (Figure 1).

The anthropometric and performance data for the participants in the acute exercise setup are shown in Table 1.

### 2.2. Differential Gene Expression Signatures between the Trained and Untrained Skeletal Muscle at Rest

**Figure 2:**
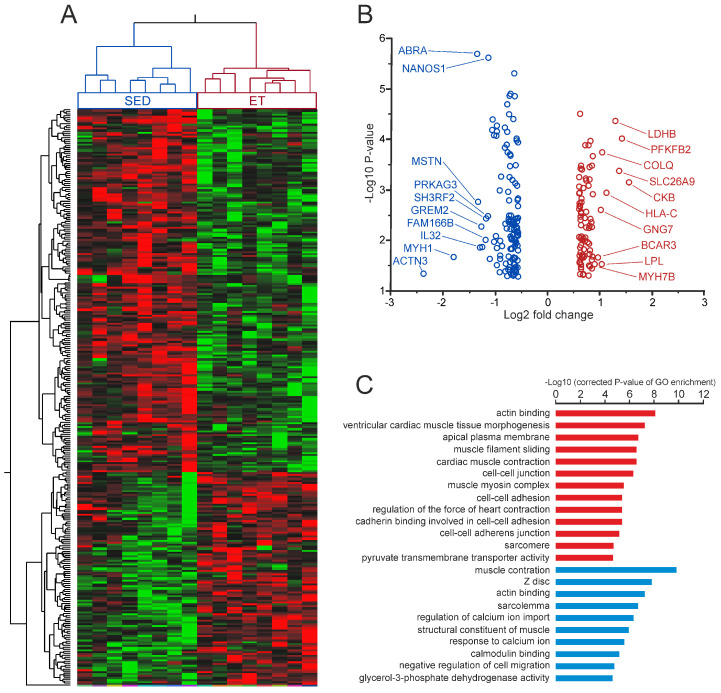
Baseline transcriptome signatures. (**A**) Heat map of differentially expressed probe sets (fold difference |≥| 1.5, *p* < 0.05) at rest in vastus lateralis muscle between endurance-trained (ET, n = 8) and sedentary (SED, n = 8) individuals. Probe sets (rows) and individuals (columns) have been ordered based on hierarchical clustering. Relative expression values are represented as a color scale according to the deviation from the average level of expression across all individuals, ranging from green for low expression to red for high expression. (**B**) Volcano plot of differentially expressed genes between ET and SED. The logarithms of the fold changes are plotted against the negative logarithm of the *p*-value to base 10. Positive Log2 fold change values represent genes that display significantly higher expression levels in the ET group (red circles), and negative values indicate increased expression in SED individuals (blue circles). The top 10 genes with highest fold change differences are highlighted for both groups. (**C**) Significantly enriched GO terms in genes with significantly higher baseline levels in ET (red) and SED (blue) individuals, respectively. GO terms were sorted based on corrected *p* values (−Log10 (Q-value) > 5).

To compare the transcriptomic landscape of the trained and untrained muscle, baseline microbiopsy samples from eight SED individuals and eight ET individuals were used for microarray gene expression profiling. By applying a fold change cut-off of |≥| 1.5 (*p* < 0.05), our microarray analysis identified a total number of 214 genes with differential baseline expression levels between the SED group and the ET group (Figure 2, Table S1).

**Figure 3:**
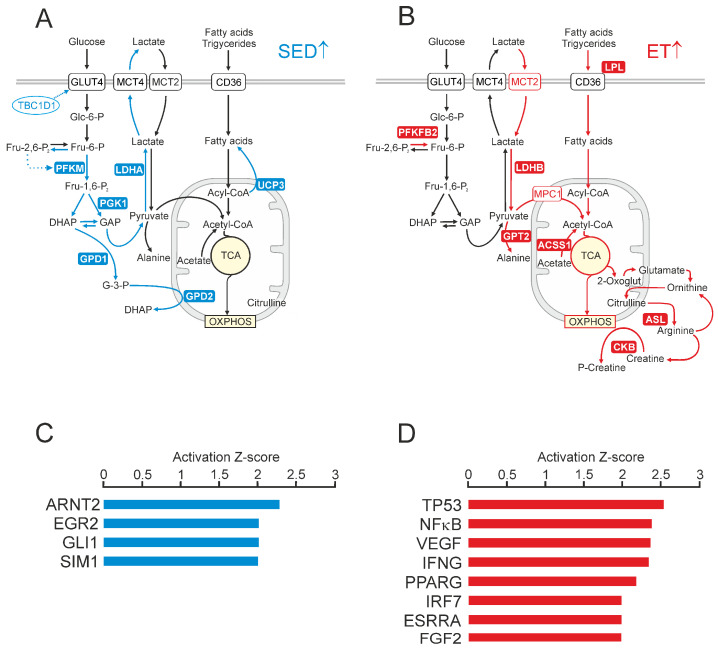
Differentially expressed metabolic genes and upstream regulators that define the basal transcriptome signature of the endurance-trained and untrained muscle. (**A**,**B**) Schematic representation of glycolytic and oxidative substrate flux with localization of key enzymes and transporters that display differential baseline gene expression levels between sedentary (SED) and endurance-trained (ET) individuals in *vastus lateralis* muscle at rest. Colors represent significant fold differences (*p* < 0.05) in the comparison of SED/ET (**A**, blue) and ET/SED (**B**, red), respectively. (**C**,**D**) Ingenuity upstream regulator analysis based on genes with significantly higher baseline expression levels in SED (**C**, blue) and ET (**D**, red) individuals, respectively. The top activated upstream regulators are shown with predicted activation Z-score ≥ 2 and a *p*-value of overlap < 0.05.

Among them, 127 genes displayed increased expression in the SED group, while 87 genes showed higher relative transcript levels in ET individuals (Figure 2B). Hierarchical clustering of differentially expressed probe sets showed that this gene signature distinguished between SED and ET individuals (Figure 2A). The *vastus lateralis* muscle is commonly characterized by a mixed fiber type composition containing both type I and type II fibers at almost equal levels in healthy, untrained individuals [26]. GO term enrichment analysis revealed that differentially expressed genes at rest were primarily attributable to cellular components and functions involved in myofibrillar structure and activity (Figure 2C). A literature search for experimentally verified fiber-type specific expression profiles within these enriched gene sets revealed a preferential up-regulation of slow-twitch (type I) muscle candidate genes in the ET group, while SED individuals showed a tendency toward higher expression of genes predominantly attributable to fast-twitch (type II) muscle fibers (Table 2). The same pattern appeared to account for differentially expressed genes involved in key metabolic pathways of substrate breakdown, with relatively higher expression levels for genes encoding glycolytic enzymes in the SED group and up-regulated oxidative metabolism genes in the ET group (Figure 3A,B).

Candidate upstream regulators that are predictively involved in determining the SED muscle phenotype included the beta subunit of hypoxia-inducible factors (HIFs) ARNT2 (aryl hydrocarbon receptor nuclear translocator 2), as well as the transcription factor (TFs) early growth response 2 (EGR2), zinc finger protein GLI1 (glioma-associated oncogene homolog 1), and class E basic helix–loop–helix protein SIM1 (single-minded family BHLH transcription factor 1) (Figure 3C). In ET muscles, we found activation signatures for upstream regulatory influence by pro-angiogenic factors, including vascular endothelial growth factor (VEGF) and fibroblast growth factor (FGF), as well as the pro-inflammatory cytokine interferon (IFN)-gamma (Figure 3D). Stimulated TF activity in the ET group was predicted for several well-known regulators of endurance exercise-dependent gene expression, including the peroxisome proliferator-activated receptor γ (PPARG), the tumor suppressor protein p53 (TP53), and the estrogen-related receptor α (ESRRA). Moreover, we also found predicted activation of candidate upstream regulators that are involved in tumor necrosis factor receptor (TNF-R)-associated factor (TRAF) signaling, namely interferon regulatory factor 7 (IRF7) and nuclear factor-kappa B (NF-κB). Significantly up-regulated targets of the NF-κB signature in the ET muscle included caspase 4 (*CASP4*), the immunoglobulin kappa light chain (*IGKC*), calcium-transporting ATPase (*ATP2A2*), the tryptophan catabolizing enzyme indoleamine 2,3-dioxygenase (*IDO1*), the kinase insert domain receptor (*KDR*) of VEGF, the Ca^2+^/calmodulin-dependent protein kinase Pim-3 (*PIM3*), as well as the tumor necrosis factor superfamily member TRAIL (*TNFSF10*).

### 2.3. Gene Expression Responses of the Trained and Untrained Skeletal Muscle Following an Acute Bout of Endurance Exercise

**Figure 4:**
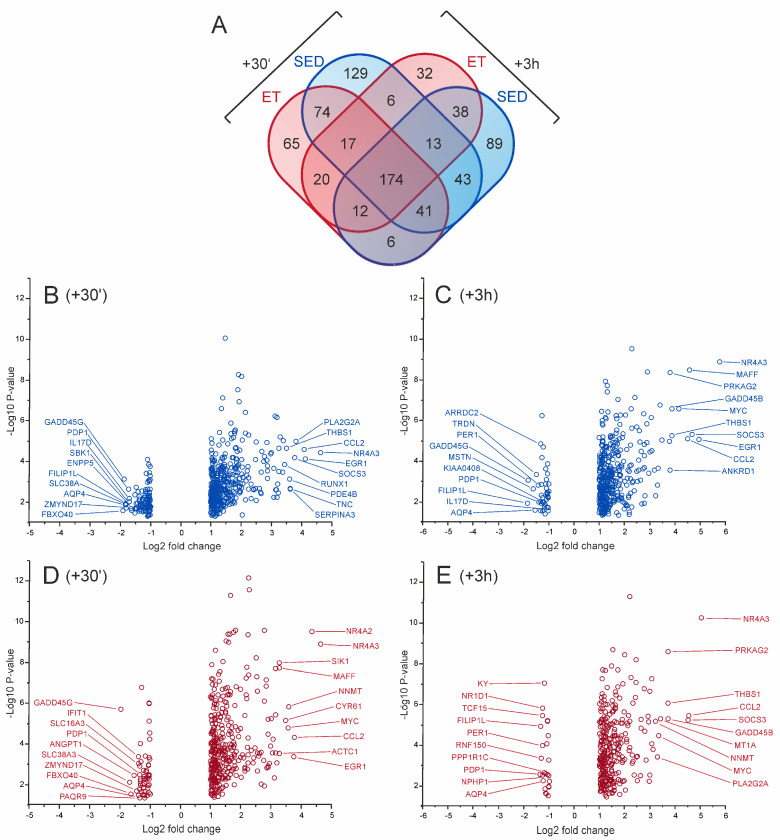
Acute exercise-provoked transcriptional responses in endurance-trained and untrained muscle. (**A**) Venn diagram showing the overlap of acute exercise-affected genes (fold change |≥| 2 versus baseline, *p* < 0.05) between sedentary (SED, blue) and endurance-trained (ET, red) individuals *in vastus lateralis* muscle at 30 min (+30′) and at 3 h (+3 h) after intense cycling exercise for 1 h. (**B**–**E**) Volcano plots for acute exercise-affected genes in SED (**B**,**C**, blue) and ET (**D**,**E**, red) individuals at +30′ (**B**,**D**) and at +3 h (**C**,**E**) after the exercise protocol. The logarithms of the fold changes are plotted against the negative logarithm of the *p*-value to base 10. Positive log2 fold change values represent genes that are significantly up-regulated by acute exercise, and negative values indicate genes that become repressed in response to exercise. The top 10 genes with highest fold change differences compared to baseline values at rest are highlighted.

To examine the impact of an acute bout of endurance exercise on skeletal muscle gene expression, all ET and SED individuals underwent 60 min of strenuous cycling exercise at a main intensity corresponding to 80% of the individual VO_2_max (including 10 min of warm-up). For both groups, this individually adjusted exercise protocol provoked comparable increases in blood lactate levels and heart rate responses (Table 1). Microbiopsy samples of the *vastus lateralis* muscle were taken at rest, 30 min (+30′) after completion of exercise, and after a recovery period of 3 h (+3 h). Overall, we found a total number of 759 genes that significantly reacted in response to exercise (fold change vs. baseline |≥| 2, *p* < 0.05), with 614 genes being up-regulated and 145 genes becoming down-regulated (Figure 4A–E; Table S1). Exercise response patterns included 600 genes (478 up, 122 down) at 30 min post exercise and 491 genes (435 up, 56 down) after 3 h of recovery.

**Figure 5:**
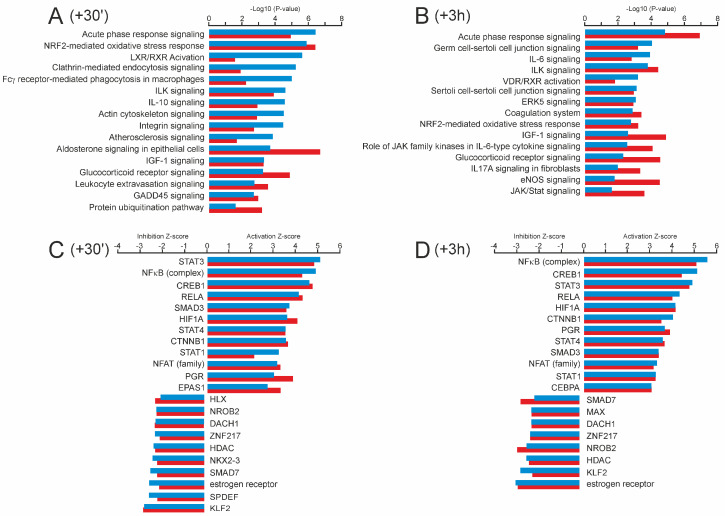
Identification of canonical pathways and upstream regulators that shape skeletal muscle response to acute exercise. (**A**,**B**) Comparison of top canonical pathways (ingenuity pathway analysis, IPA) between SED (blue bars) and ET (red bars) individuals that are enriched in exercised-affected gene sets at +30′ (**A**) and at +3 h (**B**) after exercise. Bars indicate −log *p* value (ratio of molecules present in the dataset out of all the function-related molecules). (**C**,**D**) Predicted upstream transcriptional regulators of genes that are significantly affected by exercise at +30′ (**C**) and at +3 h (**D**) in SED (blue bars) and ET (red bars) individuals. The top activated upstream regulators are shown with predicted activation Z-score |≥| 2 and a *p*-value of overlap <0.05. Positive Z-scores indicate activated factors, while negative scores indicate that a factor is inhibited.

IPA pathway analysis revealed prominent gene signatures connected to pathways of acute contractile, metabolic, oxidative, and inflammatory stress responses in both groups at both post-exercise sampling points (Figure 5A,B). Remarkably, at +30′, the untrained muscle of the SED individuals displayed higher activation scores for the nuclear receptors liver X receptor (LXR) and farnesoid X receptor (FXR), which are known to be involved in balancing lipid and glucose metabolism [40].

Potential upstream regulators positively involved in acute exercise-dependent gene regulation included several signal transducers and TFs commonly associated both with inflammatory (signal transducer and activator of transcription 1, STAT1; STAT3; STAT4; NFκB; NFκB Subunit, RELA; progesterone receptor, PGR) and regenerative processes (cAMP responsive element binding protein 1, CREB1; SMAD family member 3, SMAD3; catenin beta 1, CTNNB1), as well as with oxidative stress balance (HIF1A; endothelial PAS domain protein 1, EPAS1 (HIF2A); nuclear factor of activated T-cells, NFAT) (Figure 5C,D). Overall, acute response patterns of the trained and untrained muscles displayed considerable quantitative divergence, with only 308 genes being significantly affected at +30′ both in the ET and the SED group, and 238 genes being included in both response signatures at +3 h (fold change vs. baseline |≥| 2, *p* < 0.05).

Significant enriched GO terms for molecular functions associated with higher exercise-provoked responsiveness in the SED group (fold difference SED/ET ≥ 1.5, *p* < 0.05) included ‘transcriptional activator activity’ (*p* = 5.01 × 10^−7^) and ‘steroid hormone receptor activity’ (*p* = 1.78 × 10^−4^), with genes encoding early growth response 1 (*EGR1*), *HIF1A*, nuclear receptor subfamily 4 group A member 1 (*NR4A1*) and member 3 (*NR4A3*), AP-1 transcription factor subunit (*FOSL1*), abhydrolase domain-containing protein 2 (*ABHD2*), and retinoic acid receptor alpha (*RARA*). From the lists of exercise-affected stress signaling genes, we found six genes displaying training status-dependent differential response patterns, all of them with increased responsiveness in the untrained skeletal muscle. These genes included the transcriptional repressor *BACH1* as well as the transcriptional activator of the small MAF protein family *MAFF*, the angiotensin precursor *AGT*, and the heat shock protein family members *DNAJB4*, *DNAJC1,* and *HSP8*. In contrast, functional categories enriched in acute exercise-affected genes with higher responsiveness in the ET group (fold difference ET/SED ≥ 1.5, *p* < 0.05) were attributable to GO terms ‘phosphatidylinositol-3-kinase activity’ (*p* = 3.41 × 10^−3^) and ‘insulin receptor binding’ (*p* = 1.35 × 10^−5^), with prominent genes encoding insulin receptor substrate 2 (*IRS2*), as well as regulatory subunits 1 (*PIK3R1*) and 3 (*PIK3R3*) of phosphoinositide-3-kinase.

In total, differential expression levels between the SED group and the ET group could be observed for 188 genes at time point +30′ and for 268 genes at +3 h, with an overlap of 63 genes being included in both differential expression signatures. Considering all differentially expressed genes from both post-exercise sampling points, the most highly enriched GO term in the ET group was ‘mitochondrial matrix’ (*p* = 6.73 × 10^−20^), while in the SED group, we identified increased expression of genes primarily enriched in GO terms ‘cell growth’ (*p* = 2.03 × 10^−11^) and ‘cell proliferation’ (*p* = 1.05 × 10^−10^).

**Figure 6:**
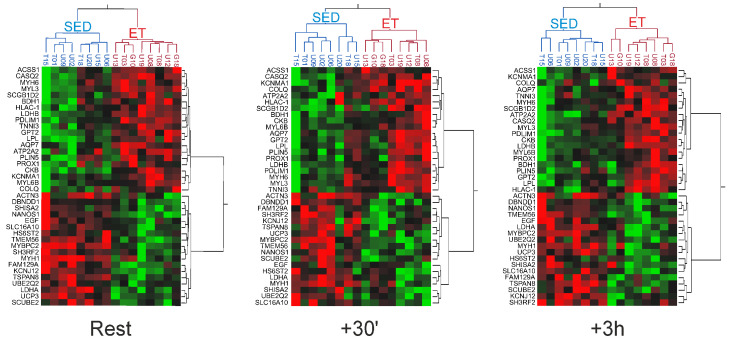
Most stable transcriptional signature of exercise-unresponsive genes that delineates the endurance-trained muscle from the untrained muscle. Heat maps depicting a stable panel of 39 genes in *vastus lateralis* muscle that retain differential expression levels (fold difference I ≥ I 1.5, *p* < 0.05) between endurance-trained (ET, red) and sedentary (SED, blue) individuals at baseline (rest) as well as in response to acute exercise (+30′, +3 h). Probe sets (rows) and individuals (columns) have been ordered based on hierarchical clustering. Relative expression values are represented as a color scale according to the deviation from the average level of expression across all individuals, ranging from green for low expression to red for high expression.

From the list of the 214 genes with differential baseline expression between the SED group and the ET group, 65 genes were also detected being differentially expressed at +30′, and 78 of these genes displayed differential expression levels at +3 h (fold difference |≥| 1.5, *p* < 0.05). To determine the most stable signature genes of the differential baseline profile, we performed cluster analysis and identified a set of 39 exercise-resilient genes (Figure 6). This stable training status-dependent profile included several well-known candidates that determine skeletal muscle architecture, contraction pattern, and metabolism, like, e.g., genes coding for muscle contractile proteins, lipoprotein lipase (*LPL*), *LDH* isoforms, or *UCP3*. In addition, we found some unexpected targets that are yet poorly defined or have not been addressed so far in connection with phenotypic skeletal muscle adaptation pattern, like the genes encoding shisa family member 2 (*SHISA2*), dysbindin domain containing 1 (*DBNDD1*), nanos C2HC-type zinc finger 1 (*NANOS1*), transmembrane protein 56 (*TMEM56*), cell growth-inhibiting protein 39 (*FAM129A*), or signal peptide CUB domain (*SCUBE2*).

In contrast to this stable gene signature, 35 genes from the differential resting expression pattern became significantly affected by the acute exercise intervention (fold change vs. baseline |≥| 2, *p* < 0.05). A list of these genes, including a detailed illustration of how they react to the exercise intervention, is given in the Supplementary Materials (Table S2). These genes obviously become epigenetically poised in the course of endurance training and represent flexible key signaling nodes of skeletal muscle plasticity that contribute both to the maintenance of long-term adaptation patterns as well as to the short-term adaptational response to exercise.

### 2.4. Expression Pattern of Selected Target Genes in the Untrained Skeletal Muscle in the Course of a Standardized Eight-Week Endurance Training Intervention

To provide independent validation of the microarray data, we included an additional cohort of 10 untrained individuals (SED-T) to absolve the 60 min cycling exercise protocol. Microbiopsy samples of the *vastus lateralis* muscle were taken at rest and at 3 h (+3 h) after completion of exercise. For confirmation of exercise-affected expression pattern by qPCR, we selected a subset of 15 candidate genes comprising targets with pronounced and moderate responsiveness, as well as targets that were considered unresponsive (fold change |≤| 2) to acute exercise. Selected candidate genes included *NR4A3*, interleukin 6 (*IL6*) and interleukin 6 receptor (*IL6R*), ZFP36 ring finger protein (*ZFP36*), TNF receptor superfamily member 12A (*TNFRSF12A*), tripartite motif-containing protein 63 (*TRIM63*), F-box protein 32 (*FBXO32*), actin-binding Rho-activating protein (*ABRA*), peroxisome proliferator-activated receptor gamma coactivator 1 alpha (*PPARGC1A*), vascular endothelial growth factor A (*VEGFA*), myostatin (*MSTN*), actinin alpha 3 (*ACTN3*), lactate dehydrogenase isoforms A (*LDHA*) and B (*LDHB*), and lipoprotein lipase (*LPL*). As shown in the Supplementary Materials (Figure S1), for 14 genes, comparisons between microarray data and qPCR analysis in the independent data set were in close agreement. In contrast to the microarray data set, the acute exercise effect on *ABRA* expression was not consistent in the independent SED-T cohort. However, it should be noted that indicated increases in the microarray cohort of SED individuals were below the fold change threshold of |≥| 2 that was applied to filter differential expression levels.

Following the acute exercise testing, all SED-T volunteers followed a standardized 8-week endurance training program, as described in detail in the Section 4. This training routine led to significant improvements in cardiorespiratory fitness. The anthropometric and performance data for the participants in the training intervention program are shown in Table 3.

**Figure 7:**
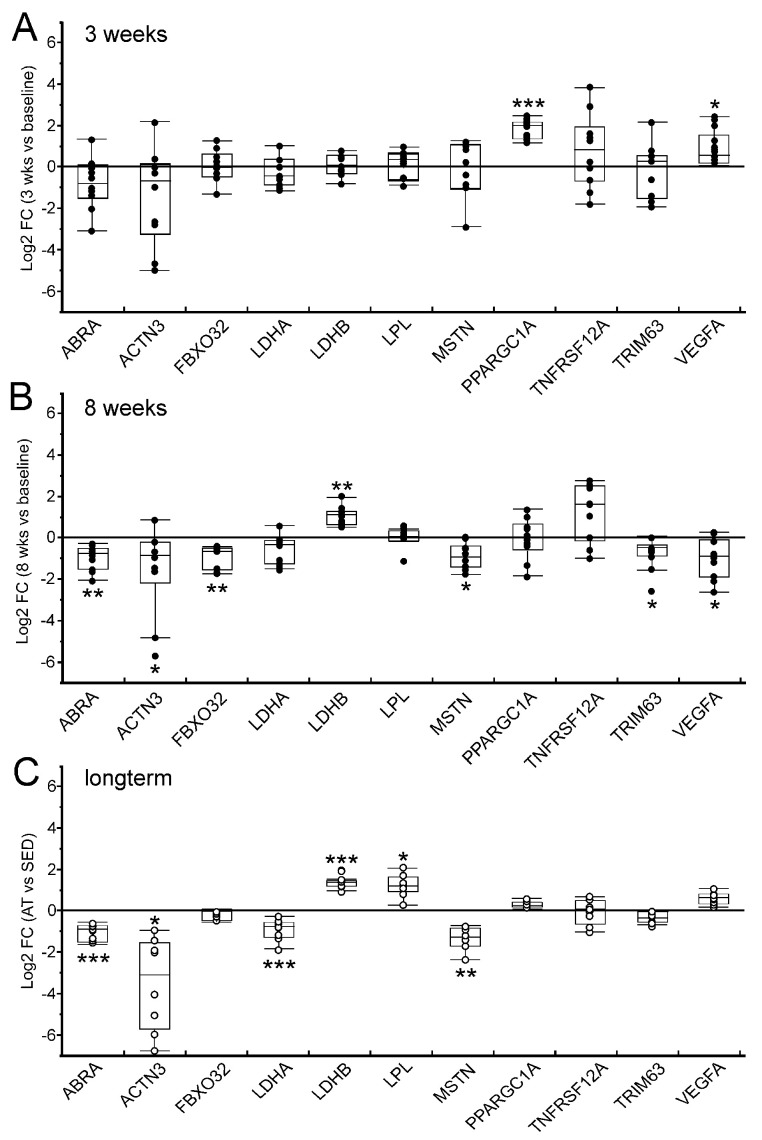
Muscular changes in basal transcript levels after 3 and 8 weeks of endurance training. Box plots represent Log2-transformed fold changes (FCs) in relative mRNA levels of selected target genes in *vastus lateralis* muscle. Short-term ((**A**), 3 wks) and medium-term ((**B**), 8 wks) changes in basal gene expression in the course of a standardized endurance exercise training program were assessed by qPCR from sedentary individuals (SED-T; n = 10) and compared to individual pre-training levels (baseline). Long-term adaptational changes in skeletal muscle transcriptional activity are reflected by the differential baseline expression pattern between endurance-trained (ET; n = 8) and sedentary (SED; n = 8) individuals that were derived from microarray data (**C**). * *p* < 0.05, ** *p* < 0.01, *** *p* < 0.001.

Basal expression profiles of *vastus lateralis* muscle for 11 target genes were analyzed by qPCR after 3 weeks (3 wks) of training as well as at the end of the training session (8 wks) and compared to pre-training baseline levels (rest) (Figure 7A,B). Selected genes included long-term-adaptation markers (*ABRA*, *ACTN3*, *LDHA*, *LDHB*, *LPL*, *MSTN*) as well as master regulators of oxidative energy metabolism (*PARGC1A* and *VEGFA*) and known markers of skeletal muscle atrophy (*FBXO32*, *TNFRSF12A*, *TRIM63*). To assess to what extent the untrained muscle became shifted toward an endurance-trained phenotype by the 8-week training intervention, we compared training-provoked changes in baseline expression levels with the differential baseline expression profile of ET and SED individuals from our microarray data (Figure 7C). This way, we aimed to decipher the short-term and long-term effects of endurance training on skeletal muscle expression patterns. As shown in Figure 7A, after 3 weeks of training, for most target genes, we observed only minor or inconsistent changes in basal transcriptional expression. However, we found a significant transcriptional up-regulation of the key metabolic regulator *PPARGC1A* gene as well as of the angiogenic marker *VEGFA*, indicating the initiation of training-induced metabolic and vascular adaptation programs. After 8 weeks of endurance exercise training, *PPARGC1A* transcript levels had ceased to baseline expression, whereas the differential expression markers *ABRA*, *ACTN3*, *LDHB*, and *MSTN* had approached the endurance-trained expression profile (Figure 7B). We further observed a significant reduction in transcript levels for the atrophy-related marker genes *FBXO32* and *TRIM63*. In contrast, robust alteration of baseline transcriptional activity for the metabolic markers *LDHA* and *LPL* was not yet manifested by 8 weeks of training.

## 3. Discussion

In the present study, we provide a comprehensive list of differentially expressed genes that distinguish the basal transcriptome pattern of human *vastus lateralis* muscle from healthy young ET and SED individuals. Most of the differentially expressed transcripts encode proteins that mediate muscle contraction and force production, ion homeostasis, as well as metabolic substrate utilization and oxidation, which mirror the major functional differences between slow- and fast-twitch muscle fibers. In line with our recent in-depth analysis of training-induced proteomic patterns in *vastus lateralis* muscle [21], we identified transcriptional signatures that reflect an improved oxidative capacity of the endurance-trained skeletal muscle. Approximately 55% of the genes that we found differentially expressed in the basal skeletal muscle transcriptome of our young ETs have also been reported by Lindholm et al. to become significantly affected in young sedentary individuals following 3 months of endurance training intervention [41], and about 30% were also found differentially expressed in middle-aged men and women as a result of decades-long training in a recent cross-sectional study by Chapman et al. [42]. Adaptations in metabolic substrate flux can exemplarily be illustrated by a shift in the LDH isoenzymes expression pattern that was consistently observed in all three studies. Basal down-regulation of *LDHA* and up-regulation of *LDHB* in the course of endurance training indicate an increased lactate threshold, augmented mitochondrial pyruvate fueling, and enhanced mitochondrial respiration [43]. Remodeling of the tetrameric LDH complex has previously been shown to be highly correlated with exercise performance and skeletal muscle oxidative capacity [44,45]. Intriguingly, we could show that 8 weeks of regular endurance exercise did already suffice to modulate skeletal muscle *LDH* isoform baseline expression, with *LDHB* becoming significantly up-regulated at the end of the training intervention.

It has been demonstrated that the transcriptional coactivator PGC-1α (*PPARGC1A*), a nodal regulator of mitochondrial biogenesis [46], is required for elevated *LDHB* transcription [44,47], whereas the *LDHA* promoter region contains the consensus sequences for, and is known to become regulated by, the TF HIF1α [48]. Consistently, in the skeletal muscle of SED individuals, we identified a signature of enhanced baseline upstream regulator activity for the nuclear translocator ARNT2 and the basic helix–loop–helix TF SIM1. ARNT2 forms functional heterodimers with HIF1α and with SIM1 to drive, i.e., enhanced transcription of glycolytic enzymes, like *PGK1* and *LDHA* [49], as well as expression of epidermal growth factor receptor (*EGFR*) and its ligand *EGF* [50,51], as observed in the untrained muscle of SED individuals.

EGF-EGFR signaling has recently emerged as an important and highly conserved regulatory pathway of fast-twitch skeletal muscle fiber type determination, and reciprocally, EGFR blockade both in vitro and in vivo appears as a suitable tool to promote a slow-twitch muscle phenotype [52]. Transient EGFR signaling, however, is also crucially important for skeletal muscle regeneration, and redox regulation of EGFR offers protection against oxidative stress and provides increased resistance to hypoxia [53,54]. EGF may also contribute to the expression of UCP3 [55], a mitochondrial inner membrane transport protein that has been subject to much controversial debate in connection with skeletal muscle physiology. UCP3 belongs to the UCP subfamily of mitochondrial anion carrier proteins and is commonly considered to play important roles in oxidative stress resistance and fatty acid metabolism; however, its mechanisms of action as well as its physiological role in the exercising muscle are still enigmatic [56,57]. In line with our data, a suppressive effect of endurance training on skeletal muscle *UCP3* expression has been described by Russell et al., with a more pronounced impact on type I fibers [58], and UCP3 content of *vastus lateralis* muscle has been proposed to reversely correlate with cycling efficiency in trained as well as untrained individuals [59]. On the other hand, overexpression of UCP3 in muscles of mice offers protection against high-fat diet-induced insulin resistance [60], increases fatty acid oxidation, reduces oxidative stress levels, and augments the effects of endurance training on whole-body energy expenditure and fiber type characteristics [61]. Mouse studies that are based on large genetic or environmental perturbations, such as knockout mice, overexpression models, or extremely high cholesterol diets, are invaluable to understanding the basic properties of isolated key signaling modules but do not necessarily allow an adequate understanding of how these factors are integrated into distinct networks under physiological conditions. Moreover, it can lead to mistaken conclusions if singular factors with pleiotropic functions are attributed with beneficial or detrimental characteristics.

One prominent representative is the TF NF-κB, which is commonly considered a “bad guy” in muscle physiology and is known to mediate severe muscle wasting in age and disease [62]. However, activation of NF-κB signaling via the alternative, or non-canonical, pathway has also been demonstrated to trigger mitochondrial biogenesis and to improve the metabolic capacity of skeletal muscle [63,64]. In the baseline differential expression pattern from ET individuals, we not only detected activation signatures for commonly approved transcriptional mediators of skeletal muscle endurance phenotype, like PPARG, TP53, and ESRRA [13,65,66,67,68], but also found indications for enhanced NF-κB activity. Although we cannot distinguish whether this reflects canonical or non-canonical signaling, increased expression of the anti-inflammatory enzyme *IDO* may hint at the latter [69]. Moreover, the non-canonical NF-κB pathway is known to become activated through particular TNF receptor family members [70], like the sheddable type II transmembrane protein TRAIL (*TNFSF10*) that we found being transcriptionally elevated in the ET muscle at rest. Again, TRAIL also seemingly shows a Janus-faced nature as, on the one side, it has been linked to muscle fiber damage in conditions of inflammatory myopathies [71] but also has been shown capable of reducing inflammation, improving insulin responsiveness, and attenuating metabolic abnormalities in diabetes [72,73].

While the precise contribution of NF-κB signaling to the baseline gene expression pattern remains elusive, in the exercising muscle, we clearly could depict NF-κB together with the TF HIF1α as being among the major drivers of the acute exercise-provoked transcriptional stress response, both in SED as well as in ET individuals, as illustrated by a significant enrichment of related pathways in the exercise-provoked gene signatures. Both TFs have previously been shown to become activated in skeletal muscle during acute exercise, accumulate in the nucleus, and associate with chromatin at their target sites [51,74,75]. Moreover, there is increasing evidence that these two signaling pathways are intimately linked and coordinately act together in shaping exercise-provoked myofibrillar stress signaling to cope with acute shortages in energy and oxygen supplies, accumulation of reduced oxygen intermediates, perturbations in intracellular calcium levels, enforced mechanical strain at the contractile apparatus, and increased release of stress hormones [75,76,77]. In the exercising muscle, NF-κB and HIF1α cooperate with multiple signaling molecules and pathways [75,78]. Nodes of crosstalk are mediated by other transcriptional regulators for which we found enriched downstream target signatures within the acute exercise-provoked expression patterns, such as members of the STAT family of TFs, the cAMP-response element binding protein (CREB), and the β-catenin signaling cascade (CTNNB1). These transcriptional regulators either directly interact with HIF and NF-κB subunits or affect the expression of common stress-responsive target genes [79,80,81].

The most prominent class of molecules that act as initiators of the acute exercise-provoked stress response are reactive oxygen species (ROS). Physiological triggering of ROS by exercise has been shown essential for normal skeletal muscle contractility and subsequent adaptation processes, while, on the other hand, excess or chronic exposure to ROS is detrimentally implicated in conditions of skeletal muscle wasting induced by disuse, aging, or disease [82,83]. Oxidative stress initiates cellular defense mechanisms that centrally involve the antioxidant-response-element signaling pathway via activation of nuclear factor erythroid 2-related factor 2 (NRF2), a TF that controls genes encoding protective antioxidants and phase II detoxification enzymes [84]. Consistently, we detected increased exercise-induced expression of NRF2 target genes, including mitochondrial superoxide dismutase (*SOD2*), which converts highly cytotoxic superoxide radicals to hydrogen peroxide, and heme oxygenase-1 (*HMOX1*), which provides bilirubin to scavenge and degrade hydrogen peroxide. Moreover, we observed acute transcriptional induction of inhibitor of differentiation (ID) proteins (*ID1*, *ID3*), a class of transcriptional regulators that have recently been found to promote antioxidant gene expression through the regulation of small MAF protein expression [85]. While increased expression of *ID*s was restricted to the acute exercise-induced transcriptional response, with similar profiles in ET and SED individuals at 30 min post exercise, up-regulated expression of the small MAF transcript *MAFF* was prolonged until 3 h post exercise, with significantly higher levels in the SED group. Small MAF proteins are capable of forming repressive homodimers as well as transcriptionally active MAF-NRF2 heterodimers that are required for efficient NRF2 binding to its target promoter sites [86]. Reciprocally, small MAF proteins can also heterodimerize with the TF BACH1, a negative regulator of antioxidant-response-element-mediated gene expression, thereby promoting active resolution of the oxidative stress response. Also of interest is that the transcriptional expression of *BACH1* in the *vastus lateralis* muscle became acutely triggered by our exercise protocol and remained elevated after 3 h of recovery. As a general theme in acute stress responses, simultaneous co-triggering of self-regulatory gene expression networks is a necessary means for rapid and flexible adjustment of cellular homeostasis to ensure proper modulation as well as timely resolution of, in the long-term, potentially harmful stress-provoked signaling cascades [87].

A primary limitation associated with the current study is that our post-exercise samples constitute a compilation of individual “snapshots” from very dynamic gene expression processes with response kinetics that may individually vary not only as a result of the training history but also due to numerous genetic and environmental factors. Despite significant quantitative differences in transcript numbers and expression intensities, the transcriptome programs activated by the intense exercise bout appeared broadly similar among ETs and SEDs, and we could not detect a clear training status-dependent theme in the acute exercise response patterns. Largely similar acute transcriptional responses have also been reported by Rubenstein et al. when muscle samples from active/endurance-trained and sedentary older adults were compared after 40 min of submaximal cycling [88]. In contrast, a substantial quantitative as well as qualitative effect of the training status on the acute exercise-provoked transcriptional response was demonstrated by Furrer et al. when quadriceps muscles of mice that had undergone 4 weeks of endurance training were compared with sedentary controls [46]. The authors noted that training status-dependent shifts in peak expression have a major impact on post-exercise transcriptomes at a given timepoint, indicating that it is rather the kinetic pattern of gene induction and repression than differences in specific gene expression signatures that distinguish the trained from the untrained muscle in its acute transcriptional response to exercise. Necessarily, endurance exercise training studies in mice cannot be directly translated to humans due to profound species-specific differences in evolutionary background, morphometry, physiology, life history, and skeletal muscle fiber composition and adaptation potential. However, in congruence with Furrer et al. [46], we observed that acute exercise-responsive genes only poorly overlapped with the persistent transcriptomic changes in the trained muscle, mainly affecting genes that were up-regulated in the untrained baseline profile. Apparently, the basal transcriptomic output that characterizes the trained muscle is only a final echo of the epigenetic alterations that become imprinted in the course of prolonged endurance training [13,41,46,89], where probably each bout of exercise is remembered by distinct chromatin marks, altered DNA methylation patterns, changes in spatial chromatin structure, and/or diverse non-coding RNA species [8]. In this way, gene loci that shape and determine skeletal muscle plasticity can reversibly be altered to reside either in an active, poised, bivalent, or suppressed state. Not surprisingly, most stably up-regulated or down-regulated genes distinctly distinguished the trained from the untrained muscle, irrespective of the muscular activation status, encoded structural proteins of the contractile apparatus, membrane transport proteins, and metabolic enzymes with known fiber-type preferences. In addition, we also found some unexpected marker genes that are yet poorly defined. Here, *SHISA2*, an endoplasmic reticulum (ER)-localized protein with up-regulated expression in the untrained muscle, is of particular interest as it has recently been shown to regulate myogenesis and satellite cell fusion [90].

From the many factors that have been implied in exercise adaptation, the transcriptional coactivator PGC-1α (encoded by the gene *PPARGC1A*) has been shown indispensable for normal transcriptional muscle plasticity, both in controlling basic pathways of the acute stress response as well as in shaping the physiological long-term training response [7,10,46]. In our training study, after 3 weeks of regular endurance exercise, we observed a significant up-regulation of *PPARGC1A*, alongside increased expression of the angiogenic factor *VEGFA*, that preceded the remodeling of the basal transcriptome landscape toward the ET phenotype, which became apparent after 8 weeks. This supports the model that muscular adaptation to endurance training is not altogether a gradual process but rather relies on an adaptive threshold system that allows constant assessment of whether the benefits to blunt the homeostatic threats imposed by frequently repeated exercise bouts outweigh the costs of structural and biochemical remodeling of skeletal muscle architecture and metabolism. Most importantly, it cannot be emphasized enough that three training sessions per week over only 2 months suffice not only to significantly improve the overall cardiorespiratory fitness of untrained individuals but also to substantially shift the skeletal muscle transcriptome toward an endurance phenotype and to significantly diminish the expression of atrophy-related marker genes.

Findings from the present study should be interpreted with the following considerations in mind. The skeletal muscle biopsy samples analyzed in this study only represent a small part of a mixed-fiber type muscle and also include varying amounts of other cell types. We also recognize that expression levels between transcript and protein are not necessarily correlated. This study lacked a parallel proteome analysis and a quantitative characterization of fiber type composition. Finally, as the current study involved only small cohorts of young, healthy males, our findings should be verified in other populations using larger sample sizes.

In summary, we provide an extensive examination of the transcriptional patterns that distinguish the endurance-trained muscle from the untrained muscle in young adult males, both at baseline as well as in response to an acute exercise challenge. We aimed to predict genes and pathways that are important to determine and maintain skeletal muscle phenotype and metabolism. Our data offer a glimpse at the fascinating complexity of the multifaceted mechanistic framework that allows our muscles to cope with the homeostatic challenges of acute exercise and to remodel their transcriptional landscape in the course of prolonged exercise training. A better understanding of the cellular and molecular mechanisms involved in exercise adaptation is fundamentally important to take full advantage of the enormous benefits that exercise training offers in disease prevention and therapy.

## 4. Materials and Methods

### 4.1. Study Participants and Phenotyping

Healthy young males were recruited and classified based on their self-reported physical activity as described in detail previously [21]. To meet the inclusion criteria for the endurance-trained (ET) group, participants had to engage in regular endurance exercise training of >5 h per week for at least 5 years. The sedentary group (SED) comprised subjects that habitually spent less than 2 h per week with moderate physical activity. Potential volunteers underwent preliminary baseline screening, including lactate diagnostics and maximal bicycle spiroergometry. Participants undertook a graded exercise test to volitional exhaustion on a cycle ergometer (Ergoselect 200, Ergoline GmbH, Bitz, Germany) for the determination of maximal oxygen uptake (VO_2_max), peak power output, and the lactate thresholds [lactate turning point 1 (LTP1) and lactate turning point 2 (LTP2)]. Before starting the test, resting blood pressure and capillary blood lactate concentrations [La−] were measured. The test began with a 2 min resting period on the bike, followed by 25 W step increments every 3 min, starting at 50 W until task failure. [La−] was analyzed (Biosen S-Line, EKF diagnostics, Barleben, Germany) by collecting capillary blood samples from the right earlobe during the last 20 s of each stage and right after volitional exhaustion. Blood pressure was taken again at 100 W and immediately after volitional exhaustion. Heart rate and electrocardiogram were constantly recorded throughout the test (12-channel PC ECG, Custo med GmbH, Ottobrunn, Germany). Breath-by-breath pulmonary gas exchange and ventilation were measured using a metabolic cart (MetaLyzer, CORTEX Biophysics GmbH, Leipzig, Germany). Calibration was performed before each test, following the manufacturer’s instructions. The course of the VO_2_ was interpolated using a spline procedure and smoothed. The plateau phase, which occurs when the O_2_ kinetics level off, was used as a criterion for determining the VO_2_max. Breath-by-breath data were averaged over 20 s. The power (in Watt) performed at the time of 100%, 80%, and 60% VO_2_max was recorded. To be included in this study, ET subjects had to exhibit an aerobic capacity of >57 mL/min/kg BW, while the VO_2_max upper limit for the SED group was set at 49 mL/min/kg BW. The study protocol was approved by the local ethics committees of the University of Tübingen, the Justus-Liebig University Giessen, and the University of Ulm. Each participant underwent a medical screening and had to give informed written consent.

### 4.2. Acute Exercise Setup and Training Intervention

Within 4 weeks from the initial performance diagnostic, the study participants followed the acute exercise protocol, which consisted of 60 min of high-intensity cycling exercise on a cycle ergometer (Ergometrics 800 S, Ergoline) at a main power requiring 80% of the individual VO_2_max (including 10 min of initial warm-up cycling at 60% VO_2_max) (Figure 1). All exercise sessions were conducted in the morning (9:00 a.m.) after an overnight fast and after receiving a standardized breakfast (8:00 a.m.).

In a follow-up study, additional SED volunteers (SED-T) were recruited to absolve an 8-week training program that consisted of 3 supervised endurance exercise sessions per week on a cycle ergometer. Before and after the training period, all participants underwent lactate diagnostics and maximal spiroergometry to determine individual anaerobic threshold (IAT) and VO_2_max. Exercise training intensity was monitored by heart rate (HR) and manually adjusted by the cycle ergometer load. The training protocol consisted of 15 min warm-up followed by 65 min of continuous cycling in the target HR range. Training intensity was progressively increased every 2 weeks, starting with a power output corresponding to 60% of the individual VO_2_max, followed by 65%, 70%, and finally 75% in weeks 7 and 8.

The anthropometric and performance data for the participants in the acute exercise setup and in the training intervention program are shown in Table 1 and Table 3, respectively.

### 4.3. Muscle Biopsies and Microarray Analysis

All muscle biopsies were taken under sterile conditions from the lateral portion of the *m. vastus lateralis* of the right leg using a fine-needle punch biopsy technique (Plus Speed; Peter Pflugbeil, Zorneding, Germany) under local anesthesia (1% Meaverin). Baseline samples of the resting muscle were obtained 24 h before the acute exercise protocol was started. Post-exercise biopsies were taken at 30 min and at 3 h after cessation of acute cycling exercise (Figure 1). Snap-frozen muscle biopsy samples were homogenized with a TissueRuptor homogenizer (Qiagen, Hilden, Germany), and total RNA was extracted and purified using Rneasy Fibrous Tissue Mini kit and Rneasy Micro Kit (Qiagen), according to the manufacturer’s recommendations. RNA integrity was assessed with Agilent 2100 Bioanalyzer (Agilent Technologies, Waldbronn, Germany), and only high-quality RNA (RNA integrity number > 7) was submitted to RNA profiling using Human Genome U219 (HG-U219) microarray platform (Affymetrix, Santa Clara, CA, USA). Preparation of biotin-labeled complementary RNA probes, hybridization, and scanning of arrays was carried out according to the manufacturer’s protocols by the Microarray Facility Tübingen (MFT Services, Tübingen, Germany). Scanned images (DAT files) were transformed into intensities (CEL files) by GCOS (Affymetrix). The raw intensity values were background corrected, log2 transformed, quartile normalized, and summarized using the RMA algorithm in Genomics Suite 6.6 (Partek). The data are deposited in the NCBI Gene Expression Omnibus (http://www.ncbi.nlm.nih.gov/geo/) and are accessible through GEO Series accession number GSE250122. Differentially expressed genes were determined using the package “limma” (version 3.24.14), which analyzes the expression data by fitting linear models and determines statistical significance with moderated t-statistics [91]. The significance threshold for differential expression at baseline and after exercise intervention between and within groups was set to a minimum log2 fold change of 0.6 (fold change |≥| 1.5) for unpaired samples and a minimum absolute log2 fold change of 1 (fold change |≥| 2) for paired samples together with *p* < 0.05. Principal component analysis (PCA) scatter plots were used to visualize patterns in the gene expression data (Figure S2). Full lists of differentially expressed probe sets are provided in the Supplementary Materials (Table S1). Enriched gene ontology (GO) terms in differentially expressed genes were identified using InCroMap [92]. Differentially expressed genes were further analyzed for significant enrichment in affected targets of known upstream regulators (Fisher’s exact test) by use of IPA^®^ Upstream Regulator Analysis (IPA; Ingenuity Systems, Redwood City, CA, USA). Heatmaps and dendrograms were created with JMP statistical software (Version 11; SAS Institute, Cary, NC, USA) by hierarchical cluster analysis of normalized log2 expression intensities using Ward’s method. Volcano plots were applied to visualize fold changes and *t*-test values of the comparisons between exercise groups and time points.

### 4.4. Quantitative RT-PCR Analysis

Microarray data were further validated in the independent cohort of sedentary individuals (SED-T) that followed the acute cycling protocol prior to participating in the 8-week training program. Skeletal muscle biopsies were analyzed by quantitative RT-PCR (qPCR) at baseline, 3 h after acute exercise, during (3 wks) the training program, and 2 days after the last training session (8 wks). Frozen biopsy samples were homogenized (4000 rpm, 2× 30 s) with Precellys beads (VWR, Darmstadt, Germany) in TRIzol reagent (ThermoFisher Scientific, Waltham, MA, USA) using a BeadBug™ microtube homogenizer (Benchmark Scientific, Inc., Edison, NJ, USA), and total RNA was isolated according to the manufacturer’s protocol. RNA was further purified using the RNeasy Micro Kit (Qiagen) as per manufacturer’s instructions, including an on-column DNase treatment step. First-strand cDNA was synthesized from 0.3 µg of total RNA, using random primers and M-MLV reverse transcriptase RNase H Minus, Point Mutant (Promega, Mannheim, Germany). Quantitative RT-PCR was performed from diluted cDNA (1:10) on an iCycler single-color detection system (BioRad, München, Germany) with SsoFast EvaGreen Supermix (BioRad) in a total reaction volume of 15 μL. Detailed information for each target-specific primer pair is provided in the Supplementary Materials (Table S3). Primers were used at a final concentration of 0.3 µM. All samples were run in duplicate. PCR was performed for 40 cycles at 95 °C for 5 s and at 59 °C for 15 s after initial incubation at 95 °C for 30 s. PCR product specificity was confirmed by melting curve analysis. Five candidate reference genes (TATA-box binding protein, *TBP*; ribosomal protein L13a, *RPL13A*; large ribosomal protein P0, *RPLP0*; beta-2-microglobulin, *B2M*; glyceraldehyde-3-phosphate dehydrogenase, *GAPDH*) were analyzed for most stable expression pattern using RefFinder [93]. The geometric mean of the three most stably expressed reference genes (*TBP*, *RPL13A*, *RPLPO*) was applied to transform threshold cycles of target genes into normalized expression values using the delta Ct method. Fold differences were calculated using the delta-delta Ct method [94].

### 4.5. Statistical Analysis

Physiological parameters, qPCR data, and subsets of microarray data were analyzed using JMP statistical software (Version 11; SAS Institute, Cary, NC, USA). Differences in subject characteristics and physical performance outcomes between and within study groups were determined via paired and unpaired *t*-tests. Relative gene expression levels and fold changes were log2 transformed. Repeated-measurement analysis of variance was used to compare differences regarding changes in expression levels at different timepoints. *p*-values were corrected for multiple testing using the Benjamini–Hochberg method. Data were considered significant with *p*-values of less than 0.05.

## Figures and Tables

**Table 1:** Physical characteristics and performance data of sedentary (SED) and endurance-trained (ET) subjects that participated in the acute exercise trial. Values represent means ± SD. BMI, body mass index; VO_2_max, maximal aerobic capacity; ***, significantly different compared with SED group (*p* < 0.001).

	Physical Characteristics			Acute Exercise Performance		
	Age (Years)	BMI (kg/m^2^)	VO_2_max (mL/min/kg)	Power Output at 80% VO_2_max (Watt)	Heart Rate at End (min^−1^)	Blood Lactate at End (mmol/L)
SED (n = 8)	22.6 ± 2.8	23.7 ± 3.7	40.3 ± 7.6	140 ± 40	180 ± 7	6.8 ± 3.2
ET (n = 8)	25.4 ± 3.8	22.9 ± 2.0	67.2 ± 8.9 ***	260 ± 60 ***	179 ± 9	7.4 ± 3.8

**Table 2:** List of genes significantly enriched in GO terms related to muscle structure and activity (GO:0030018, GO:0008307, GO:0005859, GO:0030049, GO:0006936, GO:0030017, GO:0042383) among differentially expressed genes between endurance-trained (ET) and sedentary (SED) individuals in vastus lateralis muscle at rest. Fold differences are given in the comparison of ET/SED with genes that showed relatively higher expression levels in the ET group (ET↑) and in the comparison of SED/ET when baseline levels were higher in the SED group (SED↑). Candidates with known patterns of fiber-type-preferential expression are indicated by ‘slow’ for type I fibers and ‘fast’ for type II fibers. N/D, no data available.

Gene Symbol	Gene Title	Protein Function	Up-Regulated in	Fold Difference	*p*-Value	Preferential Fiber Type	Ref.
*MYH7B*	Myosin, heavy chain 7B, cardiac muscle, beta	Motor protein of myofilaments	ET↑	1.9	2.7 × 10^−2^	slow	[27]
*ATP2A2*	ATPase, Ca++ transporting, cardiac muscle, slow twitch 2	Sarcoplasmic reticulum calcium pump	ET↑	1.8	2.4 × 10^−3^	slow	[28]
*TNNI3*	Troponin I type 3 (cardiac)	Regulator of actin–myosin interaction	ET↑	1.8	4.7 × 10^−3^	slow	[29]
*KCNMA1*	Potassium large conductance calcium-activated channel, subfamily M, alpha member 1	Regulator of muscle membrane potential	ET↑	1.8	2.6 × 10^−3^	N/D	
*CASQ2*	Calsequestrin 2 (cardiac muscle)	Calcium buffer in the sarcoplasmic reticulum	ET↑	1.8	9.2 × 10^−4^	slow	[30]
*MYL6B*	Myosin, light chain 6B, alkali, smooth muscle, and non-muscle	Constituent of myofibrillar apparatus	ET↑	1.7	5.1 × 10^−4^	slow	[31]
*MYH6*	Myosin, heavy chain 6, cardiac muscle, alpha	Motor protein of myofilaments	ET↑	1.6	1.8 × 10^−2^	slow	[27]
*PROX1*	Prospero homeobox 1	Regulator of contractile protein gene expression	ET↑	1.6	8.8 × 10^−3^	slow	[32]
*MYL3*	Myosin, light chain 3, alkali; ventricular, skeletal, slow	Constituent of myofibrillar apparatus	ET↑	1.5	2.8 × 10^−3^	slow	[27]
*MYOM3*	Myomesin family, member 3	Structural constituent of the sarcomere	ET↑	1.5	6.9 × 10^−4^	slow	[33]
*SLMAP*	Sarcolemma associated protein	Regulator of excitation–contraction coupling	SED↑	1.5	6.7 × 10^−3^	slow	[33]
*SORBS2*	Sorbin and SH3 domain containing 2	Assembly and maintenance of myofibrils	SED↑	1.6	3.5 × 10^−2^	N/D	
*PPP3CA*	Protein phosphatase 3, catalytic subunit, alpha isozyme	Calcineurin; regulator of muscle mass	SED↑	1.6	9.6 × 10^−6^	fast	[34]
*CAV3*	Caveolin 3	Integral membrane component of caveolae	SED↑	1.6	4.7 × 10^−2^	fast	[35]
*NEDD4*	Neural precursor cell expressed, developmentally down-regulated 4	Mediator of skeletal muscle atrophy	SED↑	1.6	4.1 × 10^−3^	N/D	
*MYL5*	Myosin, light chain 5, regulatory	Constituent of myofibrillar apparatus	SED↑	1.6	3.6 × 10^−3^	fast	[31]
*KCNJ12*	Potassium inwardly rectifying channel, subfamily J, member 12	Stabilization of the resting membrane potential	SED↑	1.6	8.0 × 10^−3^	N/D	
*VCL*	Vinculin	Structural component of costameres	SED↑	1.6	1.3 × 10^−2^	slow/fast	[33]
*DTNA*	Dystrobrevin, alpha	Structural component of costameres	SED↑	1.6	1.3 × 10^−2^	fast	[33]
*KIAA1161*	KIAA1161	Putative regulator of myogenesis	SED↑	1.7	3.1 × 10^−2^	fast	[36]
*MYBPC2*	Myosin binding protein C, fast type	Regulator of myofilament activation and relaxation	SED↑	1.7	2.3 × 10^−5^	fast	[37]
*FHL2*	Four and a half LIM domains 2	Putative regulator of sarcomere assembly	SED↑	1.7	2.7 × 10^−3^	slow	[38]
*HOMER1*	Homer homolog 1 (Drosophila)	Regulator of transient receptor potential channel activity	SED↑	1.7	8.7 × 10^−4^	N/D	
*LMOD1*	Leiomodin 1 (smooth muscle)	Regulator of actin filament length	SED↑	1.9	2.1 × 10^−2^	N/D	
*ABRA*	Actin-binding Rho activating protein	Sarcomere-binding transcriptional regulator	SED↑	2.0	8.3 × 10^−5^	N/D	
*ATP2B2*	ATPase, Ca++ transporting, plasma membrane 2	Plasma membrane calcium pump	SED↑	2.2	2.1 × 10^−2^	N/D	
*MYH1*	Myosin, heavy chain 1, skeletal muscle, adult	Motor protein of myofilaments	SED↑	3.5	1.9 × 10^−2^	fast	[36]
*ACTN3*	Actinin, alpha 3	Structural component of sarcomeric Z line	SED↑	5.3	4.3 × 10^−2^	fast	[39]

**Table 3:** Baseline physical characteristics of sedentary subjects (SED-T; m; age, 27 ± 2.5) before (pre) and after (post) the 8-week exercise routine. Values represent means ± SD. BMI, body mass index; IAT, individual anaerobic threshold; VO_2_max, maximal aerobic capacity; ***, significantly different compared with individual pre-training level (*p* < 0.001).

	Weight (kg)	BMI (kg/m^2^)	IAT (Watt/kg)	VO_2_max (mL/min/kg)
pre (n = 10)	79.5 ± 13.9	25.3 ± 4.4	1.6 ± 0.5	36.1 ± 6.3
post (n = 10)	79.2 ± 14.0	25.3 ± 4.3	2.0 ± 0.3 ***	43.7 ± 6.4 ***

## Data Availability

Microarray data are deposited in the NCBI Gene Expression Omnibus (http://www.ncbi.nlm.nih.gov/geo/) and are accessible through GEO Series accession number GSE250122.

## Supplementary Materials

The following supporting information can be downloaded at https://www.mdpi.com/article/10.3390/ijms25052881/s1.

## Abbreviations

ACSS1, acyl-CoA synthetase short-chain family member 1; ARNT2, aryl hydrocarbon receptor nuclear translocator 2; ASL, argininosuccinate lyase; CD36, fatty acid translocase; CKB, creatine kinase brain; EGR2, early growth response 2; ESRRA, estrogen-related receptor alpha; FGF2, fibroblast growth factor 2; GLI1, glioma-associated oncogene family zinc finger 1; GLUT4, glucose transporter type 4; GPD1, glycerol-3-phosphate dehydrogenase 1 (soluble); GPD2, glycerol-3-phosphate dehydrogenase 2 (mitochondrial); GPT2, glutamic pyruvate transaminase; IFNG, interferon gamma; IRF7, interferon regulatory factor 7; LDHA, lactate dehydrogenase A; LDHB, lactate dehydrogenase B; LPL, lipoprotein lipase; MCT2 (SLC16A7), monocarboxylate monocarboxylic acid transporter 2; MCT4 (SLC16A4), monocarboxylate monocarboxylic acid transporter 4; MPC1 (BRP44L), mitochondrial pyruvate carrier 1; NFκB, nuclear factor kappa B (complex); PDHB, pyruvate dehydrogenase, beta; PFKFP2, 6-phosphofructo-2-kinase/fructose-2,6-biphosphatase 2; PFKM, phosphofructokinase, muscle; PGK1, phosphoglycerate kinase 1; PPARG, peroxisome proliferator activated receptor gamma; SIM1, single-minded family BHLH transcription factor 1; TBC1D1, TBC1 domain family member 1; UCP3, uncoupling protein 3; TP53, tumor protein P53; VEGF, vascular endothelial growth factor.
