# Supplementary material for: The Acute, Short-, and Long-Term Effects of Endurance Exercise on Skeletal Muscle Transcriptome Profiles
Source: Int J Mol Sci. 2024 Mar 1;25(5):2881. doi: 10.3390/ijms25052881 (PMC10932090; doi:10.3390/ijms25052881)
Supplement: Supplementary file 1 [file ijms-25-02881-s001.zip › Supplementary data IJMS.pdf]

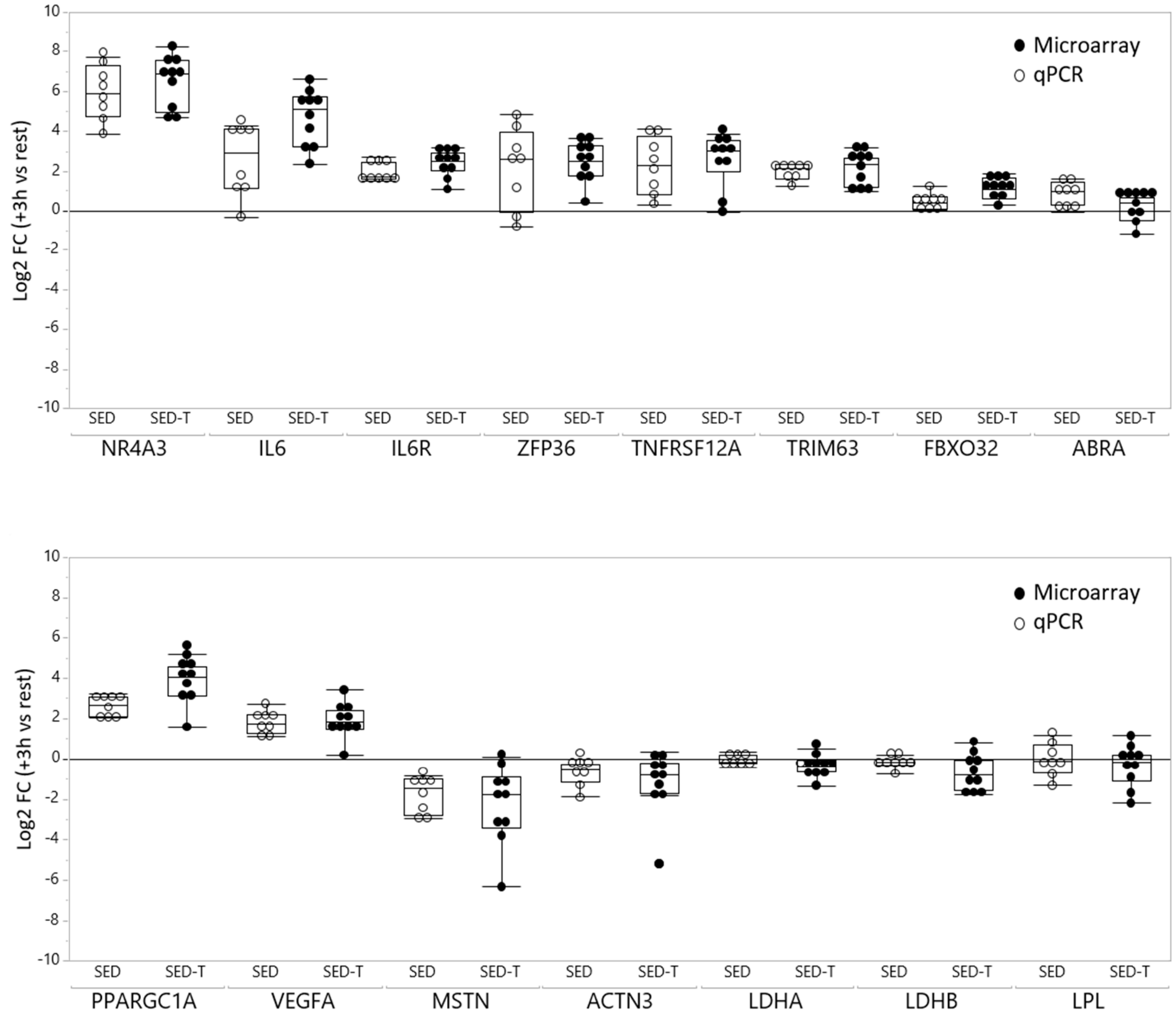

Figure S1.

Independent validation of microarray data from sedentary individuals (SED, n = 8, open circles) for exercise-responsiveness of 15 selected target genes in a second cohort of untrained individuals (SED-T, n =10, filled circles) using qPCR. Box plots represent Log2 transformed fold changes in mRNA levels from *vastus lateralis* muscle at 3 hours (+3h) after intense cycling exercise in comparison to baseline levels at rest (+3h/rest).

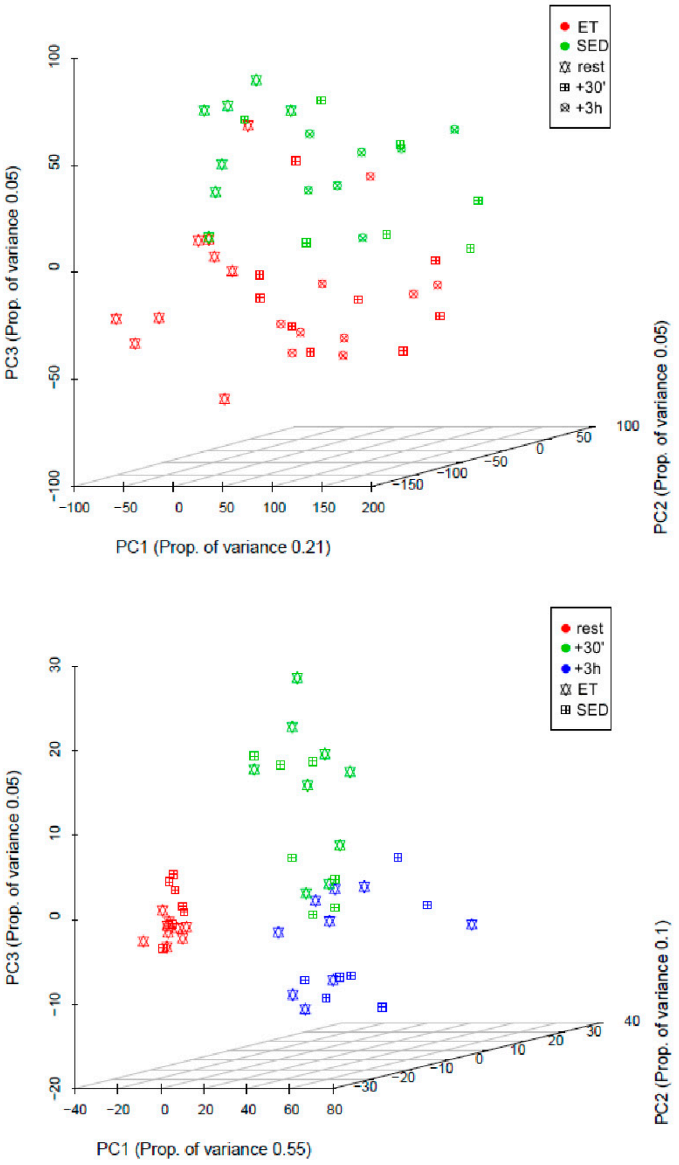

Figure S2.
Principal component analysis (PCA) scatter plots of gene expression profiles of *vastus lateralis* muscle samples from endurance-trained (ET) and sedentary (SED) individuals at baseline (rest), at 30 min (+30’) and at 3 hours (+3h) after intense cycling exercise.

Table S2 (next page)

List of genes among differentially expressed genes at rest (fold difference I≥I 1.5, P < 0.05) between endurance-trained (ET) and sedentary (SED) individuals in *vastus lateralis* muscle that were significantly affected by acute exercise in at least one group at any post-exercise time point (fold change I≥I 2, P < 0.05). Mean fold differences in baseline expression levels (rest) are given in the comparison ET/SED with genes that showed relative higher expression levels in the ET group (ET↑), and in the comparison SED/ET when baseline levels were higher in the SED group (SED↑). Gene expression changes in response to acute exercise are given as mean fold changes compared to baseline expression at 30 min (+30') after completion of exercise, and after a recovery period of 3 hours (+3h). Positive fold change values indicate increased expression after exercise (↑) while negative fold change values (↓) indicate decreased expression in the respective group.

		Baseline expression pattern			Exercise response pattern					
		Rest			+30'			+3h		
Gene symbol	Gene title	Up-regulated in	Fold difference	P-value	Affected in	Fold change vs rest	P-value	Affected in	Fold change vs rest	P-value
PFKFB2	6-phosphofructo-2-kinase/fructose-2,6-biphosphatase 2	ET↑	2.7	7.3E-05				ET↑<br>SED↑	2.0<br>3.5	1.4E-05<br>1.4E-03
SLC38A1	Sodium-coupled neutral amino acid transporter 1	ET↑	1.6	1.3E-02				ET↑<br>SED↑	2.6<br>2.8	1.3E-04<br>1.0E-03
PPP1R1C	Protein phosphatase 1, regulatory (inhibitor) subunit 1C	ET↑	1.7	4.5E-02	ET↓<br>SED↓	-2.3<br>-2.1	2.0E-03<br>9.6E-04	ET↓	-2.3	2.5E-03
CEACAM1	Carcinoembryonic antigen-related cell adhesion molecule 1 (biliary glycoprotein)	ET↑	1.5	4.3E-03				ET↑<br>SED↑	2.2<br>2.0	3.9E-05<br>4.3E-06
CASP4	Caspase 4, apoptosis-related cysteine peptidase	ET↑	1.5	8.1E-03	ET↑<br>SED↑	2.7<br>2.3	5.6E-04<br>1.3E-03	ET↑<br>SED↑	2.9<br>2.0	2.4E-04<br>4.4E-03
PIM3	Pim-3 oncogene	ET↑	1.6	3.1E-04				SED↑	2.5	2.0E-08
BCAR3	Breast cancer anti-estrogen resistance 3	ET↑	1.9	2.0E-02				SED↑	3.0	9.9E-04
AQP7	Aquaporin 7	ET↑	1.8	3.2E-03	SED↓	-2.1	2.6E-02			
TNFSF10	Tumor necrosis factor (ligand) superfamily, member 10	ET↑	1.5	1.1E-02	ET↓	-2.4	1.5E-07			
CYP4V2	Cytochrome P450, family 4, subfamily V, polypeptide 2	ET↑	2.1	1.8E-02	ET↓	-2.1	1.4E-02			
PAQR9	Progestin and adipoQ receptor family member IX	ET↑	1.8	3.3E-02	ET↓	-2.6	1.2E-03			
FILIP1L	Filamin A interacting protein 1-like	SED↑	1.6	8.5E-03	ET↓<br>SED↓	-2.4<br>-3.6	1.0E-02<br>1.3E-02	ET↓<br>SED↓	-2.4<br>-3.6	1.0E-02<br>1.3E-02
MSTN	Myostatin	SED↑	2.6	1.4E-03	SED↓	-2.9	4.2E-03	ET↓<br>SED↓	-2.0<br>-3.1	6.8E-03<br>2.5E-03
DNAJB4	DnaJ (Hsp40) homolog, subfamily B, member 4	SED↑	1.6	6.0E-04	ET↑<br>SED↑	2.1<br>2.0	5.6E-07<br>4.0E-04			
RND3	Rho family GTPase 3	SED↑	1.7	3.5E-02				ET↑<br>SED↑	2.3<br>2.1	2.6E-03<br>1.1E-04
KY	Kyphoscoliosis peptidase	SED↑	1.6	5.0E-02	ET↓<br>SED↓	-2.0<br>-2.0	9.7E-07<br>1.7E-02	ET↓<br>SED↓	-2.3<br>-2.2	9.4E-08<br>1.4E-03
PMP22	Peripheral myelin protein 22	SED↑	1.5	2.7E-02				ET↑<br>SED↑	3.8<br>2.4	9.7E-08<br>9.4E-04
DTNA	Dystrobrevin, alpha	SED↑	1.6	1.3E-02	ET↑<br>SED↑	7.5<br>6.3	1.2E-03<br>3.8E-03	ET↑<br>SED↑	4.0<br>4.3	1.8E-02<br>1.7E-02
MLF1	Myeloid leukemia factor 1	SED↑	1.7	1.4E-04	SED↓	-2.2	2.3E-03			
PLAGL2	Pleiomorphic adenoma gene-like 2	SED↑	2.0	4.0E-05				SED↓	-2.4	5.9E-07
SLC38A4	Sodium-Coupled neutral amino acid transporter 4	SED↑	1.9	4.3E-04				SED↓	-2.1	4.6E-03
GLRX	Glutaredoxin (thioltransferase)	SED↑	1.9	4.0E-02	SED↑	2.0	3.5E-02			
IRX3	Iroquois homeobox 3	SED↑	2.3	8.6E-05	SED↓	-2.0	4.7E-03			
ATP2B2	ATPase, Ca++ transporting, plasma membrane 2	SED↑	2.2	2.1E-02	SED↓	-2.5	1.9E-02			
LMOD1	Leiomodin 1	SEDS↑	1.9	2.1E-02	SED↓	-2.4	8.5E-03	SED↓	-2.1	2.4E-02
XPO4	Exportin 4	SED↑	1.7	9.8E-03	SED↓	-2.1	1.1E-02			
PRKAG3	Protein kinase, AMP-activated, gamma 3 non-catalytic subunit	SED↑	2.2	2.8E-03	SED↓	-2.1	2.6E-02			
ABRA	Actin-binding Rho activating protein	SED↑	2.6	1.3E-06	ET↑	4.4	8.5E-07	ET↑	2.5	1.5E-05
TSC22D2	TSC22 domain family, member 2	SED↑	1.7	1.4E-05	ET↑	2.2	1.1E-06	ET↑	2.3	4.1E-07
CHMP1B	Chromatin modifying protein 1B	SED↑	1.7	4.4E-04	ET↑	2.3	1.8E-06	ET↑	2.3	1.1E-06
GPD1	Glycerol-3-phosphate dehydrogenase 1 (soluble)	SED↑	1.6	4.7E-02	ET↓	-2.1	3.8E-03			
LITAF	Lipopolysaccharide-induced TNF factor	SED↑	1.6	9.9E-03	ET↑	2.0	3.0E-04	ET↑	2.1	1.1E-04
IL32	Interleukin 32	SED↑	2.5	1.2E-02	ET↑	2.3	1.7E-02			
PKNOX1	PBX/knotted 1 homeobox 1	SED↑	1.5	1.0E-02	ET↑	2.0	3.5E-04			
CHST3	Carbohydrate (chondroitin 6) sulfotransferase 3	SED↑	1.7	5.0E-03	ET↑	2.1	2.7E-04			

Table S3. List of primers for qPCR analysis

Gene symbol	Gene name	Forward primer	Reverse primer	Amplicon length (bp)	Primer efficiency
ABRA	Actin Binding Rho Activating Protein	CCTTGCCCTCCCAGGTAAAC	CCCTTTCAAGTTGCCCACTG	86	1.85
ACTN3	Actinin alpha 3	CGCAGGACATCAACACCAAG	CGGAGCCTCTCGTTTACCTG	106	1.90
B2M	Beta-2-Microglobulin	TTTCTGGCCTGGAGGCTATC	CGGATGGATGAAACCCAGAC	117	1.90
FBXO32	F-Box Protein 32	GACAAAGGGCAGCTGGATTG	TTATTGGCAGTGCACGGATG	149	1.90
GAPDH	Glyceraldehyde-3-Phosphate Dehydrogenase	TCAACAGCGACACCCACTCC	TGAGGTCCACCACCCTGTTG	126	1.95
IL6	Interleukin 6	GCCAGAGCTGTGCAGATGAG	TGGGTCAGGGGTGGTTATTG	90	1.85
IL6R	Interleukin 6 receptor	CAGCTGGGACTGTGCACTTG	AGCCTTTGTCGTCAGGGATG	134	1.90
LDHA	Lactate Dehydrogenase A	GGAAAGGCTGGGAGTTCACC	TCCACTCCATACAGGCACACTG	85	1.90
LDHB	Lactate Dehydrogenase B	TCCAGGATTCATCCCGTGTC	CGGGCATTGAGGATACATGG	92	1.95
LPL	Lipoprotein Lipase	TTGGAGAAGCTATCCGCGTG	TCGTGGGAGCACTTCACTAGC	72	1.85
MSTN	Myostatin	ATGACGATTATCACGCTACAACG	TGGGTTTTCCATCCACTTGC	84	2.00
NR4A3	Nuclear receptor subfamily 4 group A member 3	CAAGCCTTAGCCTGCCTGTC	GTGGGCTCCAGAGCCTGTC	143	1.90
PPARGC1A	PPARG Coactivator 1 Alpha	TCCCACACACAGTCGCAGTC	CCTTGGGGTCATTTGGTGAC	91	1.90
RPL13A	Ribosomal Protein L13a	CTCAAGGTCGTGCGTCTGAAG	GTGGCTGTCACTGCCTGGTAC	94	2.00
RPLP0	Ribosomal Protein Lateral Stalk Subunit P0	TCAACGGGTACAAACGAGTCC	CAGATGGATCAGCCAAGAAGG	96	1.95
TBP	TATA-Box Binding Protein	GATCAAACCCAGAATTGTTCTCC	AGCCATTACGTCGTCTTCCTG	143	1.90
TNFRSF12A	Tumor necrosis factor receptor superfamily member 12A	AGAGAAGTTCACCACCCCCATA	GGCAGAGACTGGCTCTAGAATG	141	1.95
TRIM63	Tripartite Motif Containing 63	GAGGATTCCCGTCGAGTGAC	CCAGGATGGCATACAACGTG	88	1.90
UCP3	Uncoupling Protein 3	TGCTGAGGTGGTGACCTACG	CAAAGTGGCAGGGGAAGTTG	83	1.95
VEGFA	Vascular Endothelial Growth Factor A	AAAACACAGACTCGCGTTGC	ATCTGGTTCCCGAAACCCTG	126	1.90
ZFP36	Zinc finger protein 36	ACTTCAGCGCTCCCACTCTC	GTCCCTCCATGGTCGGATG	129	1.85
